# Access to alcohol and tobacco retail outlets and its associated factors among residents of Bangkok, Thailand

**DOI:** 10.1186/s12889-026-27646-0

**Published:** 2026-05-02

**Authors:** Nongnuch Jindarattanaporn, Salakjit Chuenchom

**Affiliations:** Institute for Population and Social Research, Mahidol University, Salaya Campus, Nakhon Pathom, Thailand

**Keywords:** Alcohol, Tobacco, Purchasing, Retail access, Thailand

## Abstract

**Background:**

Alcohol and tobacco use remain major public health concerns in urban settings where retail availability is widespread. This study examined associations between sociodemographic factors, retail access, and alcohol and tobacco purchasing among urban residents in Bangkok.

**Methods:**

A cross-sectional survey was conducted among 1,000 Thai youth and adults aged 18–60 years using a multi-stage sampling approach with convenience-based household recruitment in the final stage. Alcohol purchasing in the past 12 months and tobacco purchasing in the past 30 days were assessed as binary outcomes. Sociodemographic characteristics and access to retail outlets were collected using a structured questionnaire, allowing multiple responses for outlet access. Associations were examined using Pearson’s chi-square test or Fisher’s exact test.

**Results:**

Alcohol and tobacco purchasing were common and were significantly associated with sex, age group, religion, marital status, and educational attainment. Purchasing of both alcohol and tobacco was more commonly reported among males than females and among participants aged 26–40 years compared to other age groups. Monthly income and type of residential community were associated with alcohol purchasing only. Access to physical retail outlets, particularly convenience stores and local community shops, was associated with purchasing of both alcohol and tobacco products. Access to online retailers was significantly associated with tobacco purchasing but not with alcohol purchasing.

**Conclusions:**

Alcohol and tobacco purchasing among urban residents in Bangkok was associated with sociodemographic factors and retail access, highlighting the importance of retail-focused public health policies.

## Background

Alcohol and tobacco use remain major contributors to preventable morbidity and mortality worldwide, accounting for a substantial burden of non-communicable diseases (NCDs) and related social harms [1, 2]. Beyond individual choice, increasing evidence highlights the importance of structural and environmental determinants, particularly retail access, in shaping alcohol and tobacco purchasing and use [3, 4]. Ease of access through physical proximity, outlet density, and emerging online channels plays a critical role in normalising consumption and sustaining demand [5, 6].

International evidence shows that greater retail access to physical outlets, especially convenience stores and small community shops is associated with higher levels of alcohol and tobacco purchasing and consumption [7–10]. High outlet density has been linked to earlier initiation, heavier use, and reduced effectiveness of age-based sales restrictions, prompting several high-income countries to adopt zoning regulations, licensing systems, and outlet density controls as core public health strategies [11, 12].

Online retail environments have further expanded access beyond physical outlets. Studies suggest that online platforms may facilitate tobacco purchasing through convenience and weak age verification, particularly among younger and digitally engaged populations [13, 14]. In contrast, evidence for online alcohol purchasing is more limited and context-specific [15]. In Thailand, despite strict regulation of tobacco marketing and a ban on e-cigarettes, online tobacco sales via social media and e-commerce platforms remain widespread, indicating persistent regulatory gaps in digital retail access [16].

Thailand enforces a minimum legal purchasing age of 20 years for alcohol and tobacco and restricts promotional practices [17, 18]. However, alcohol consumption among young people has increased [19] alongside the rapid expansion of convenience stores, modern retail chains, and e-marketplaces in urban areas, particularly Bangkok [10, 20, 21]. Despite the clear policy relevance of retail access, empirical evidence examining how physical and online access shapes alcohol and tobacco purchasing in urban settings in low- and middle-income countries (LMICs) remains gaps in the literature [22, 23]. Existing studies have rarely examined alcohol and tobacco purchasing simultaneously, particularly in urban settings in low- and middle-income countries (LMICs) [22, 23]. In addition, limited evidence is available on how different types of retail access, including physical outlets and online channels, are associated with purchasing behaviours within the same population [24]. Evidence from Bangkok, where retail environments are rapidly expanding and diversifying, is especially scarce [25]. Addressing these gaps is important for informing context-specific public health and regulatory strategies [26].

This study examines alcohol and tobacco purchasing among youth and adults in Bangkok, focusing on retail access to physical and online channels. By providing access-focused evidence from an urban LMIC context, the study aims to inform retail access-oriented regulatory and enforcement strategies in urban public health policy.

## Methods

This quantitative cross-sectional study used a multi-stage sampling approach to survey Thai youth and adults aged 18–60 years living in Bangkok, Thailand. A total of 1,000 participants were recruited from multiple districts and community settings across the city.

### Population and sample size

Population data were obtained from the Bangkok population and housing registration statistics (2024), stratified by age and sex. The population comprised 3,366,080 individuals aged 18–25 years and 2,865,036 individuals aged 26–60 years [27]. The sample size was calculated using Yamane’s formula: n = N / [1 + N(e²)], where *n* is the required sample size, N is the population size, and *e* is the margin of error. A margin of error of 5% (e = 0.05) was applied. Degrees of freedom were not used in this calculation. Based on the total population, the initial minimum sample size was approximately 400 participants [28]. To account for an anticipated response rate of 80%, the sample size was adjusted to 500 participants. To enable subgroup analyses by age group (youth aged 18–25 years and adults aged 26–60 years) and to ensure adequate statistical power and representation across groups, the final sample size was increased to 1,000 participants.

### Sampling frame and sampling procedure

The study employed a multistage area-based sampling approach combined with convenience recruitment at the household level. Bangkok was first divided into six geographical zones: central, northern, southern, eastern, northern Thonburi, and southern Thonburi [29]. Districts were then selected using simple random sampling proportionate to the number of districts within each zone. Within selected districts, communities were classified into informal settlements, suburban communities, urban communities, high-rise communities, and special communities. Informal, suburban, and urban communities were randomly selected, while high-rise and special communities were excluded due to access constraints [30].

Within each selected community, participants were recruited through door-to-door visits using convenience sampling. Households that declined participation were skipped, and neighbouring households were approached until the required number of participants was achieved. Five individuals aged 18–25 years and five individuals aged 26–60 years were recruited per community.

### Questionnaire development and validation

The questionnaire was newly developed for this study, drawing on multiple sources to ensure conceptual relevance and content coverage. Consumer behaviour theory [31] informed the overall framework and guided the identification of key constructs related to purchasing behaviour, including access, availability, and decision-making factors. Items on alcohol and tobacco purchasing were adapted from previously published national and international studies [21, 32–36], while selected sociodemographic and household variables were based on established measures used in national surveys conducted by the National Statistical Office of Thailand [37, 38]. The instrument comprised five sections: food and beverage purchasing behaviour, alcohol purchasing, tobacco purchasing, sociodemographic characteristics, and household characteristics [39]. Key domains, including retail access, product types, and reasons for purchasing, were derived from the literature and aligned with the study objectives. The present analysis focused on alcohol and tobacco purchasing.

Content validity was assessed by three experts in food and nutrition, alcohol, and tobacco using the Index of Item–Objective Congruence (IOC), with items achieving IOC values ≥ 0.50 retained [40]. The overall IOC score was 0.81, indicating satisfactory content validity [40]. Reliability was evaluated through a pilot test involving 30 individuals with characteristics similar to the target population. Internal consistency, assessed using Cronbach’s alpha, was acceptable for the alcohol section (α = 0.74) and good for the tobacco section (α = 0.93) [41].

### Variables and measurement

#### Independent variables

Independent variables included sociodemographic characteristics and retail access. Sociodemographic variables comprised sex, age group, religion, marital status, educational attainment, monthly income, and residential community type.

Retail access was defined based on participants’ self-reported purchasing from different types of retail outlets within the specified recall periods (past 12 months for alcohol and past 30 days for tobacco). This measure reflects behavioural access rather than objective indicators such as outlet density, geographic proximity, or travel time. The outlet types included both physical outlets (e.g., convenience stores, local community shops, hypermarkets, and supermarkets) and online channels (e.g., e-commerce platforms and social media).

Retail access was operationalised as a set of binary variables (yes/no), with each variable indicating whether the participant reported purchasing from a specific type of retail outlet during the relevant recall period. Multiple responses were permitted, allowing participants to report purchasing from more than one outlet type.

#### Outcome variables

The primary outcome variables were alcohol beverage purchasing and tobacco product purchasing, assessed as binary variables (purchased vs. not purchased). Participants were classified as having purchased alcohol beverages if they reported purchasing any alcohol beverages at least once during the past 12 months preceding the survey. Tobacco product purchasing was defined as having purchased any tobacco products at least once during the past 30 days preceding the survey. Different recall periods were applied to reflect the typical frequency of purchasing behaviours for alcohol and tobacco products, consistent with previous studies [21, 32–36].

### Types of alcohol and tobacco products

Participants reported the types of alcohol beverages purchased in the past 12 months and tobacco products purchased in the past 30 days, with multiple responses permitted. Alcohol was categorised as beer, wine, spirits, or ready-to-drink beverages, while tobacco products included manufactured cigarettes, roll-your-own cigarettes, electronic cigarettes, and heated tobacco products. All product types were coded as binary variables (purchased vs. not purchased).

### Reasons for purchasing

Reasons for purchasing included spatial convenience, product availability and in-store visibility, continuous availability, digital convenience, price promotions, and home delivery services. Multiple responses were permitted. The item product availability and in-store visibility was intentionally measured as a combined construct to capture both the availability of products and their prominence within retail environments.

### Data collection

Field interviewers were trained at the Institute for Population and Social Research, Mahidol University, between August 18 and December 2, 2025. Data were collected through face-to-face interviews using a structured questionnaire administered via the Qualtrics online survey platform on tablet or mobile devices, following coordination with community leaders. This approach enabled real-time data entry, automated skip patterns, and completeness checks, thereby enhancing data accuracy and quality. Written informed consent was obtained from all participants prior to data collection.

### Data analysis

Categorical variables were summarised using frequencies and percentages. Bivariate analyses were conducted to examine associations between sociodemographic characteristics, and alcohol beverage and tobacco product purchasing. Pearson’s chi-square test was used when expected cell counts were adequate, while Fisher’s exact test was applied as appropriate, particularly in cases of sparse data or expected cell counts less than five. A two-sided p-value of < 0.05 was considered statistically significant. All analyses were performed using SPSS software. Multivariable analysis was not conducted due to data limitations, including sparse cell counts in several variables, which may result in unstable parameter estimates. Therefore, the analysis was restricted to bivariate methods. As a result, the observed associations should be interpreted with caution, as they may be subject to confounding [42].

## Results

### Sociodemographic characteristics

Table 1 summarises the sociodemographic characteristics of the study participants (*n* = 1,000). Slightly more than half of the participants were female. The mean age was in early adulthood, with the largest proportion aged 18–25 years. The age group 18–25 years includes both individuals below and above the legal purchasing age of 20 years in Thailand; therefore, findings for this group should not be interpreted as reflecting underage purchasing. The majority of participants identified as Buddhist, were single, and had attained at least secondary or higher than secondary education. Most participants reported a monthly income below 15,000 THB and resided in urban communities.

**Table 1:** Sociodemographic characteristics of study participants

Sociodemographic characteristics	All (*n* = 1,000)	
	*n*	Percent
Sex		
Male	490	49.0
Female	510	51.0
Age (years): mean (SD) = 33.17 (13.83); median = 25; range = 18–60		
18–25	503	50.3
26–40	181	18.1
41–60	316	31.6
Religion		
Buddhist	875	87.5
Muslim	125	12.5
Marital status		
Single	564	56.4
Married/ cohabiting	378	37.8
Separated/widowed/ divorced	58	5.8
Educational attainment		
Illiterate	5	0.5
Primary school	132	13.2
Secondary school	502	50.2
Higher than secondary school	361	36.1
Average monthly income (THB): mean (SD) = 14,195.2 (9,422.0); median = 13,000; range = 400–80,000		
< 15,000	542	54.2
15,000–30,000	415	41.5
> 30,000	43	4.3
Type of residential community		
Urban community	531	53.1
Suburban community	249	24.9
Informal settlement (slum)	220	22.0

### Purchase of alcohol beverages and tobacco products

Table 2 presents the prevalence of alcohol beverage and tobacco product purchasing among participants. Alcohol beverage purchasing in the past 12 months was reported by 63.5% of respondents, while tobacco product purchasing in the past 30 days was reported by 43.0%. These estimates are not directly comparable due to differences in recall periods.

**Table 2:** Prevalence of alcohol beverage and tobacco product purchasing among study participants

Product type	All (*n* = 1,000)	
	Purchased (%)	Not purchased (%)
Alcohol beverages (any type)	63.5	36.5
1. Beer	52.3	47.7
2. Brown/red spirits	33.4	66.6
3. White spirits	16.7	83.3
4. Wine coolers	17.6	82.4
5. Ready-to-drink (RTD) beverages / fruit-flavoured alcoholic drinks / cocktails	5.4	94.6
6. Grape wine / champagne / fruit wine	2.3	97.7
7. Other alcohol beverages (e.g. herbal-infused spirits / Chinese liquor)	1.5	98.5
Tobacco products (any type)	43.0	57.0
1. Manufactured cigarettes (e.g. cigarette packs, flavoured cigarettes)	40.1	59.9
2. Roll-your-own tobacco	4.6	95.4
3. Electronic cigarettes / electronic waterpipes	1.1	98.9
4. Waterpipe tobacco (e.g. hookah, shisha)	0.3	99.7
5. Other tobacco products (e.g. cigars, pipes)	0.0	100.0

### Retail access and reasons for selecting retail outlets for alcohol beverages and tobacco products

Table 3 describes the self-reported types of retail outlets used for purchasing alcohol beverages and tobacco products, as well as the reasons for selecting these outlets. For alcohol purchasing, convenience stores (54.7%) and local community shops (45.6%) were the most commonly reported outlet types. For tobacco purchasing, local community shops (26.8%) and convenience stores (22.4%) were the most commonly reported. Online retail channels were more commonly reported for tobacco (19.0%) than for alcohol (0.2%).

The most frequently reported reasons for selecting retail outlets were spatial convenience (60.1% for alcohol; 39.3% for tobacco), followed by product availability and in-store visibility. As participants could select more than one response, percentages do not sum to 100%.

**Table 3:** Types of retail outlets used for purchasing and reasons for outlet selection among alcohol and tobacco purchasers

Purchase characteristics	Percentage of purchasing	
	Alcohol beverages (any type) (*n* = 635)	Tobacco products (any type) (*n* = 430)
Types of retail outlets used for purchasing*		
Convenience stores	54.7	22.4
Local community shops / mom-and-pop stores	45.6	26.8
Hypermarkets	31.0	0.2
Supermarkets	28.0	0.0
Online retailers (e.g. Facebook Marketplace, Lazada, Shopee, TikTok Shop)	0.2	19.0
Reasons for purchasing from these outlets*		
Spatial convenience (e.g. proximity to home, residence, or workplace)	60.1	39.3
Product availability and prominent in-store display	29.6	15.5
Availability at all times	8.5	14.3
Digital convenience (e.g. ordering via mobile applications)	6.5	4.2
Price promotions (e.g. discounts, bundle offers, giveaways, lower prices than other outlets)	5.2	1.5
Home delivery services	0.9	1.3

Note: No participants reported purchasing tobacco products from supermarkets

* Participants could select more than one response for both types of retail outlets used and reasons for purchasing; therefore, percentages do not sum to 100%. Percentages are calculated based on the number of participants who reported purchasing alcohol (*n* = 635) or tobacco (*n* = 430), respectively

### Associations between sociodemographic characteristics, access to retail outlets, and alcohol beverage and tobacco product purchasing

Table 4 presents the associations between sociodemographic characteristics, retail access, and alcohol beverage and tobacco product purchasing. Alcohol beverage purchasing was significantly associated with sex, age group, religion, marital status, and educational attainment, whereas no statistically significant associations were observed for monthly income or type of residential community. Similar patterns of association were observed for tobacco product purchasing. Access to retail outlets, particularly convenience stores and local community shops, was strongly associated with both alcohol beverage and tobacco product purchasing (Fisher’s exact test, all *p* < 0.001). Online retail channels were more frequently reported among tobacco purchasers than alcohol purchasers. As these findings are based on descriptive data and self-reported measures, they should be interpreted as patterns of outlet use rather than statistically tested associations.

**Table 4:** Associations between sociodemographic characteristics, retail access, and alcohol beverage and tobacco product purchasing

Variables	Purchase of alcohol beverages All (*n* = 1,000)				Purchase of tobacco products All (*n* = 1,000)			
	*n*	Purchased (%)	Not Purchased (%)	*P*-value	*n*	Purchased (%)	Not Purchased (%)	*P*-value
Sociodemographic characteristics								
Sex								
Male	490	70.8	29.2	< 0.001	490	56.7	43.3	< 0.001
Female	510	58.5	43.5		510	29.8	70.2	
Age (years)								
18–25	503	59.8	40.2	0.010	503	39.2	60.8	0.019
26–40	181	72.4	27.6		181	50.8	49.2	
41–60	316	64.2	35.9		316	44.6	55.4	
Religion								
Buddhist	875	68.5	31.5	< 0.001	875	41.1	56.9	0.002
Muslim	125	28.8	71.2		125	56.0	44.0	
Marital status								
Single	564	62.1	37.9	0.034	564	39.0	61.0	0.005
Married/ cohabiting	378	63.2	36.8		378	46.8	53.2	
Separated/widowed/ divorced	58	79.3	20.7		58	56.9	43.1	
Educational attainment								
Illiterate	5	40.0	60.0	0.041	5	20.0	80.0	< 0.001
Primary school	132	56.3	43.2		132	54.5	45.5	
Secondary school	502	62.7	37.3		502	46.8	53.2	
Higher than secondary school	361	61.2	38.8		361	33.8	66.2	
Average monthly income (THB)								
< 15,000	542	59.6	40.4	0.019	542	42.1	57.9	0.511
15,000–30,000	415	68.4	31.6		415	44.8	55.2	
> 30,000	43	65.1	34.9		43	37.2	62.8	
Type of residential community								
Urban community	531	64.2	35.8	< 0.001	531	43.3	56.7	0.923
Suburban community	249	53.0	47.0		249	43.4	56.6	
Informal settlement (slum)	220	73.6	26.4		220	41.8	58.2	

Note: Some categories contain small cell counts and should be interpreted with caution

## Discussion

Alcohol and tobacco purchasing were common among youth and adults in Bangkok and were associated with sociodemographic characteristics. Purchasing was prevalent among both young people aged 18–25 years and adults, and was higher among men and adults aged 26–40 years. Convenience stores and local community shops were the most commonly reported sources of purchasing for both alcohol and tobacco products. Online retail channels were more frequently reported among tobacco purchasers than alcohol purchasers.

### Access to alcohol beverages and tobacco products among young people

Although Thai law prohibits the sale of alcoholic beverages to persons under 20 years of age and bans price promotions and sales incentives [43], our study found that youth aged 18–25 years in Bangkok were able to purchase alcohol and were exposed to promotional activities, which may suggest potential gaps in law enforcement. However, the age group analysed (18–25 years) includes individuals both below and above the legal purchasing age of 20 years; therefore, these findings cannot be interpreted as direct evidence of underage purchasing.

Similar minimum legal age restrictions exist in countries such as the United Kingdom, and Australia, where stronger enforcement mechanisms, including age verification and retailer penalties [44–47], have been shown to reduce underage access [48]. Likewise, despite comprehensive prohibitions on tobacco sales to persons under 20 years of age and bans on tobacco marketing in Thailand [17, 18], youth in this study were still able to purchase tobacco products and encounter promotional practices. These findings highlight persistent enforcement gaps in Thailand’s alcohol and tobacco control systems, which may indicate areas for strengthening retail monitoring and compliance measures.

### Sociodemographic differences in alcohol and tobacco purchasing

Alcohol and tobacco purchasing were common and significantly associated with sex and age group, with higher purchasing observed among men and adults aged 26–40 years. These findings are consistent with evidence from multiple countries indicating that men and older individuals are more likely to purchase and consume alcohol more frequently and in larger quantities than women and younger populations [49]. Such patterns may be related to social and cultural norms that grant men greater acceptance to consume alcohol, including public drinking, while discouraging similar behaviours among women; in some contexts, alcohol consumption may also function as a marker of masculine status and power [50]. Higher purchasing among adults aged 26–40 years may reflect greater financial stability, disposable income, and more frequent social interactions during this life stage [51]. Similar sociodemographic gradients were observed for tobacco purchasing, reinforcing evidence that men are more likely than women to smoke and that smoking behaviour often persists from adolescence into adulthood [52–55].

### Retail outlet use and purchasing patterns

This study assessed retail access based on participants’ reported use of different outlet types rather than objective measures of the retail environment such as outlet density, proximity, or spatial distribution. Therefore, the findings should be interpreted as reflecting patterns of outlet use and purchasing behaviour rather than direct evidence of the effects of retail environment characteristics. Accordingly, this study does not provide direct empirical evidence on the impact of outlet density, proximity, or zoning and licensing policies, and these factors are discussed only as part of the broader literature. The results indicate that convenience stores and local community shops were the most commonly reported outlets for purchasing alcohol and tobacco products. This pattern is broadly consistent with existing literature suggesting that widely available and easily accessible retail channels may play an important role in shaping purchasing behaviour [56, 57]. However, the present study did not directly measure outlet density, proximity, or other environmental characteristics, and therefore cannot provide direct evidence regarding the impact of such factors.

In the Thai context, retail environments are characterised by a high presence of small community shops and convenience stores in residential areas [58, 59]. International evidence shows that regulating alcohol and tobacco availability through outlet density controls, zoning, and licensing systems can reduce consumption and related harms [60]. In the United States and Australia, local governments commonly use zoning regulations and licensing caps to restrict outlet density, particularly near schools and residential areas, as a strategy to reduce alcohol- and tobacco-related harms [61–64]. The UK has implemented cumulative impact zones and licensing restrictions to limit outlet concentration in high-risk areas [65]. These findings should be interpreted as providing contextual insights rather than direct empirical support for these specific policy measures such as outlet density controls or zoning restrictions. Further research using objective measures of the retail environment is needed to examine these relationships more directly.

### Online retail access and tobacco purchasing

Online retail channels were more frequently reported among tobacco purchasers than alcohol purchasers in this study. The limited reporting of online alcohol purchasing suggests that online channels may currently play a smaller role in alcohol purchasing behaviour in this context. Previous research indicates that online channels facilitate tobacco and e-cigarette purchasing through price incentives, convenience, and discreet access, which may be particularly appealing to younger users and those seeking lower-cost or stigma-free options [66, 67]. Prior studies have also reported increasing engagement with online tobacco purchasing and have identified sociodemographic patterns associated with online tobacco use, highlighting the growing role of digital platforms in tobacco consumption behaviours [14, 66].

In Thailand, although certain tobacco products are restricted in formal retail settings [68, 69], online platforms and social media have been reported as alternative channels for tobacco-related sales and promotion [70–72]. Empirical evidence from Thailand has documented the presence of online tobacco vendors operating through e-commerce platforms and social media networks, often incorporating promotional strategies such as price discounts and delivery services, in some cases with limited age verification mechanisms [70–72]. These findings suggest that online environments may facilitate continued consumer exposure to tobacco products despite regulatory restrictions, particularly among digitally engaged populations [71, 72]. By contrast, evidence on online alcohol purchasing in Thailand remains limited. Available literature suggests that regulatory controls, age-verification requirements, and consumer purchasing practices may limit the extent to which online retail channels influence alcohol purchasing behaviour [15]. However, this relationship requires further empirical investigation in the Thai context. Overall, these findings highlight potential product-specific differences in the role of online retail environments and suggest the need for differentiated regulatory approaches addressing online tobacco commerce alongside conventional retail control measures.

This study has several limitations. First, alcohol beverage purchasing and tobacco product purchasing were assessed using different reference periods (past 12 months for alcohol and past 30 days for tobacco), which may have introduced recall bias and limited the direct comparability of purchasing behaviours between the two product types. Second, access to retail outlets was determined based on participants’ self-reported store names, which were subsequently classified into outlet categories by the research team. Given the diversity and informal nature of retail outlets in Bangkok, some degree of misclassification may have occurred, potentially influencing the observed associations. In addition, retail access in this study reflects self-reported purchasing from outlet types rather than objective environmental measures such as outlet density, proximity, or geographic accessibility. In addition, retail access in this study reflects self-reported purchasing from outlet types rather than objective environmental measures such as outlet density, proximity, or geographic accessibility.

Third, the study employed a non-probability sampling approach, including fixed quotas of 500 youth and 500 adults. As probability sampling was not used, the findings may not be fully generalisable to the broader population of Bangkok or Thailand. Furthermore, the exclusion of some community types (e.g. high-rise residential areas and certain special communities) may further limit external validity. Fourth, the cross-sectional design precludes causal inference, and the observed associations may be subject to confounding. Although sociodemographic variables and retail access were examined, multivariable logistic regression analysis was not feasible due to sparse data and quasi-complete separation in several variables. Therefore, the findings are based on bivariate analyses only and should be interpreted as unadjusted associations. Fifth, the categorisation of age groups does not allow direct identification of individuals below the legal purchasing age of 20 years. Therefore, findings related to the 18–25 year age group should not be interpreted as direct evidence of underage purchasing. Overall, findings related to retail access are based on descriptive analyses of self-reported purchasing patterns from different outlet types and should be interpreted as indicative behavioural patterns rather than causal or independent effects of the retail environment.

## Conclusions

This study provides empirical evidence from Bangkok on patterns of alcohol and tobacco purchasing in relation to self-reported retail outlet use and sociodemographic characteristics. Convenience stores and local community shops were the most commonly reported outlets for both alcohol and tobacco purchasing, while online retail channels were more frequently reported among tobacco purchasers than alcohol purchasers. These findings highlight differences in purchasing patterns across retail channels and product types and contribute to limited evidence from urban settings in Thailand. From a public health perspective, the results support the relevance of both physical and digital retail environments in alcohol and tobacco control strategies. Overall, the findings should be interpreted as descriptive associations based on self-reported data.

## Data Availability

The data that support the findings of this study are available from Institute for Population and Social Research, Mahidol University and BioThai Foundation but restrictions apply to the availability of these data, which were used under license for the current study, and so are not publicly available. Data are however available from the authors upon reasonable request and with permission of Institute for Population and Social Research, Mahidol University and BioThai Foundation.
